# DNA damage in cumulus cells generated after the vitrification of in vitro matured porcine oocytes and its impact on fertilization and embryo development

**DOI:** 10.1186/s40813-021-00235-w

**Published:** 2021-10-18

**Authors:** Alma López, Miguel Betancourt, Yvonne Ducolomb, Juan José Rodríguez, Eduardo Casas, Edmundo Bonilla, Iván Bahena, Socorro Retana-Márquez, Lizbeth Juárez-Rojas, Fahiel Casillas

**Affiliations:** 1 Biological and Health Sciences Program, Metropolitan Autonomous University, Mexico City, Mexico; 2 Department of Health Sciences, Metropolitan Autonomous University-Iztapalapa Campus, 09340 Mexico City, Mexico; 3 Genetic and Environmental Toxicology Research Unit, FES-Zaragoza-UMIEZ Campus II, National Autonomous University of Mexico, 09230 Mexico City, Mexico; 4 Department of Biology of Reproduction, Metropolitan Autonomous University-Iztapalapa Campus, Av. San Rafael Atlixco 186, Leyes de Reforma, 09340 Mexico City, Mexico

**Keywords:** Vitrification, Matured oocytes, Cumulus cells, DNA damage, Cryoprotectants, Porcine

## Abstract

**Background:**

The evaluation of the DNA damage generated in cumulus cells after mature cumulus-oocyte complexes vitrification can be considered as an indicator of oocyte quality since these cells play important roles in oocyte developmental competence. Therefore, the aim of this study was to determine if matured cumulus-oocyte complexes exposure to cryoprotectants (CPAs) or vitrification affects oocytes and cumulus cells viability, but also if DNA damage is generated in cumulus cells, affecting fertilization and embryo development.

**Results:**

The DNA damage in cumulus cells was measured using the alkaline comet assay and expressed as Comet Tail Length (CTL) and Olive Tail Moment (OTM). Results demonstrate that oocyte exposure to CPAs or vitrification reduced oocyte (75.5 ± 3.69%, Toxicity; 66.7 ± 4.57%, Vitrification) and cumulus cells viability (32.7 ± 5.85%, Toxicity; 7.7 ± 2.21%, Vitrification) compared to control (95.5 ± 4.04%, oocytes; 89 ± 4.24%, cumulus cells). Also, significantly higher DNA damage expressed as OTM was generated in the cumulus cells after exposure to CPAs and vitrification (39 ± 17.41, 33.6 ± 16.69, respectively) compared to control (7.4 ± 4.22). In addition, fertilization and embryo development rates also decreased after exposure to CPAs (35.3 ± 16.65%, 22.6 ± 3.05%, respectively) and vitrification (32.3 ± 9.29%, 20 ± 1%, respectively). It was also found that fertilization and embryo development rates in granulose-intact oocytes were significantly higher compared to denuded oocytes in the control groups. However, a decline in embryo development to the blastocyst stage was observed after CPAs exposure (1.66 ± 0.57%) or vitrification (2 ± 1%) compared to control (22.3 ± 2.51%). This could be attributed to the reduction in both cell types viability, and the generation of DNA damage in the cumulus cells.

**Conclusion:**

This study demonstrates that oocyte exposure to CPAs or vitrification reduced viability in oocytes and cumulus cells, and generated DNA damage in the cumulus cells, affecting fertilization and embryo development rates. These findings will allow to understand some of the mechanisms of oocyte damage after vitrification that compromise their developmental capacity, as well as the search for new vitrification strategies to increase fertilization and embryo development rates by preserving the integrity of the cumulus cells.

## Introduction

Oocyte vitrification has become an important tool for the improvement of assisted reproduction in humans and other mammalian species. The oocyte meiotic stage [1], the cryoprotectant agents (CPAs) selection, and the volume of the cell-storage device [2] are key factors associated with the success of vitrification. For vitrification, CPAs are used at high concentrations (16–50%), which causes detrimental effects in oocytes and compromises their further development. The toxicity and use of high CPAs concentrations have been a limiting factor for cryopreservation success. For this reason, cryoprotectant-free vitrification methods have been attempted in human spermatozoa [3] and equine oocytes [4] without success, and so far, it has never been performed in porcine. Moreover, results from previous studies demonstrated the need for CPAs [3–6], and recently new nontoxic CPAs has been proposed [7]. For oocyte cryopreservation, ethylene glycol (EG) and dimethylsulfoxide (DMSO) have been the most widely used permeable CPAs. It was reported that its use is safer than 1,2- propanediol (PROH) [8]. Somfai et al. [2] reported that the mixture of EG + propylene glycol (PG) is similar to EG + DMSO in blastocyst production after immature oocyte vitrification. Also, we reported that immature oocyte vitrification with EG + DMSO resulted in a 30% blastocyst formation [9]. Therefore, in the present study, EG + DMSO were used for metaphase II (MII) oocytes exposure or vitrification.

In humans and other mammalian species, oocytes are mostly recovered and vitrified at the MII stage [10–14] prior to in vitro fertilization (IVF) or intracytoplasmic sperm injection (ICSI). At this stage, the cumulus cells (CCs) are only removed if ICSI is performed. However, studies have reported contradictory results, some of which highlight the beneficial effects of the CCs [15] surrounding the oocyte during vitrification [16], but another study reported that the removal of these cells before vitrification improves oocyte survival and maturation [17]. CCs play several major roles in oocyte maturation and fertilization [15] and have also been proposed as oocyte quality biomarkers [18]. In this regard, a study reported that vitrified MII oocytes with CCs resulted in higher IVF rates compared to denuded oocytes [19]. It was also reported that the CCs protect MII oocytes against zona pellucida hardening and cytoplasmic damage during vitrification-warming [20]. Additionally, the CCs prevent oocyte cryodamage by preserving the structure of major oocyte organelles after vitrification [21]. These cells are firstly exposed to the CPAs, preventing osmotic shock, facilitating oocyte dehydration, and reducing oocyte damage [20]. Because of this, it was reported that the CCs viability decreases considerably after cumulus-oocyte complexes (COCs) vitrification [20, 22, 23]. In contrast, other studies support that COCs vitrification reduces CPAs penetration and increases ice crystal formation in oocytes leading to inadequate dehydration, which affects oocyte survival [24, 25]. However, information about the alterations produced by vitrification in porcine CCs is limited.

The inefficiencies in determining the quality of the oocytes are a major issue that compromises successful fertilization rates. Since the direct evaluation of oocytes by invasive methods can impair their development, the study of CCs can reflect oocytes developmental competence. It has been reported that CCs and oocytes bidirectional communication is needed for the development and functions of both cell types [26]. Oocytes influence granulosa cells development by paracrine factors, and control metabolic activities by promoting gene expression in CCs [26]. Therefore, to evaluate if vitrification is capable of generating DNA damage in the CCs is of great importance for oocyte fertilization and embryo development (ED). For this purpose, DNA fragmentation can be measured by means of the comet assay [27]. Most studies have been carried out to evaluate the effects caused by vitrification on the oocytes leaving aside the importance of the CCs [28–30]. Stachowiak et al. [31] evaluated the DNA damage using the comet assay in bovine oocytes exposed to different vitrification methods. This study suggests that the vitrification of MII oocytes resulted in considerable DNA fragmentation. Also, DNA damage in CCs generated after cryopreservation has been reported in humans [32], bovine [33], and equine [20]. It was reported that after vitrification, greater DNA damage is generated in the peripheral CCs than in the inner CCs [4]. However, in pigs, this has not yet been evaluated. Pigs are an important experimental model since this species has anatomical, biochemical, and endocrine similarities with humans [34]. Therefore, in vitro studies may suggest some of the mechanisms of damage produced by vitrification and its possible application in humans. Thus, the evaluation of the DNA integrity after vitrification in CCs will be helpful in order to find new vitrification strategies that will increase IVF and ED rates. Therefore, the aim of this study was to determine if matured porcine COCs exposure to CPAs or vitrification affects oocyte and cumulus cells viability, and if DNA damage is generated in cumulus cells, affecting fertilization and ED.

## Materials and methods

### Experimental design

Seven replicates were performed. In each replicate all experiments were performed. In vitro matured COCs with a two-four-layer of CCs [23] were randomly distributed into four groups: (1) control (no treatment); (2) hydrogen peroxide (H_2_O_2_) was used as a DNA damage-inducer [35], positive control (COCs exposed to 2.2% of H_2_O_2_ for 5 min); (3) toxicity (COCs exposed to CPAs, EG + DMSO without vitrification); and (4) Vitrification (COCs exposed to CPAs, EG + DMSO and vitrified in Cryolock, Importadora Mexicana de Materiales para Reproducción Asistida S.A. de C.V., México). After treatments, viability was evaluated in oocytes and CCs. For this, CCs were separated from oocytes by COCs mechanical denudation. The DNA damage was evaluated only in the CCs. After treatments, to determine the importance of the CCs during IVF and ED, oocytes were fertilized in the absence (denuded oocytes, − CCs) or presence (intact COCs, + CCs) of the CCs. The number of evaluated oocytes and CCs for each experiment is shown in the description of the figure captions.

### Ethics statement

This study was approved under the regulations of the Ethics Committee for the care and use of animals; Metropolitan Autonomous University-Iztapalapa Campus.

### Oocyte collection and in vitro maturation

Ovaries were collected from F1 (Landrace X Yorkshire) pre-pubertal gilts at the “Los Arcos” slaughterhouse (State of Mexico) and transported to the laboratory in 0.9% NaCl solution at 25 °C in less than 2 h. The aforementioned facility has the animal health federal law authorization number 6265375. Ovarian follicles between 3 and 6 mm in diameter were punctured to obtain the follicular fluid. Follicular contents were left to sediment and washed twice with Tyrode modified medium supplemented with 10 mM sodium lactate, 10 mM HEPES and 1 mg/mL polyvinyl alcohol (PVA) (TL-HEPES-PVA) at pH 7.3–7.4 for COCs collection. Oocytes with uniform cytoplasm surrounded by a two-four-layer compact mass of CCs were selected. COCs were washed three times in 500 μL drops of maturation medium: TCM-199 with Earle’s salts and 26.2 mM sodium bicarbonate (In Vitro, Mexico) supplemented with 0.1% PVA, 3.05 mM D-glucose, 0.91 mM sodium pyruvate, 0.57 mM cysteine and 10 ng/mL EGF, 0.5 μg/mL LH, and 0.5 μg/mL FSH. 30–40 COCs were placed in each well of a four-well dish (Thermo-Scientific Nunc, Rochester NY) containing 500 μL of maturation medium and incubated at 38.5 °C with 5% CO_2_ in air and humidity at saturation for 44 h [9]. Maturation was evaluated by the Hoechst stain only in the negative control. Oocytes were stained with 10 μg/mL bisbenzimide (Hoechst 33342) for 40 min using an epifluorescence microscope (Zeiss Axiostar) 400× magnification for observation. Oocytes with a germinal vesicle (GV) or in metaphase I (MI) were considered immature; and those in MII with the first polar body, as matured.

### Oocyte and cumulus cells viability

**Fig. 1:**
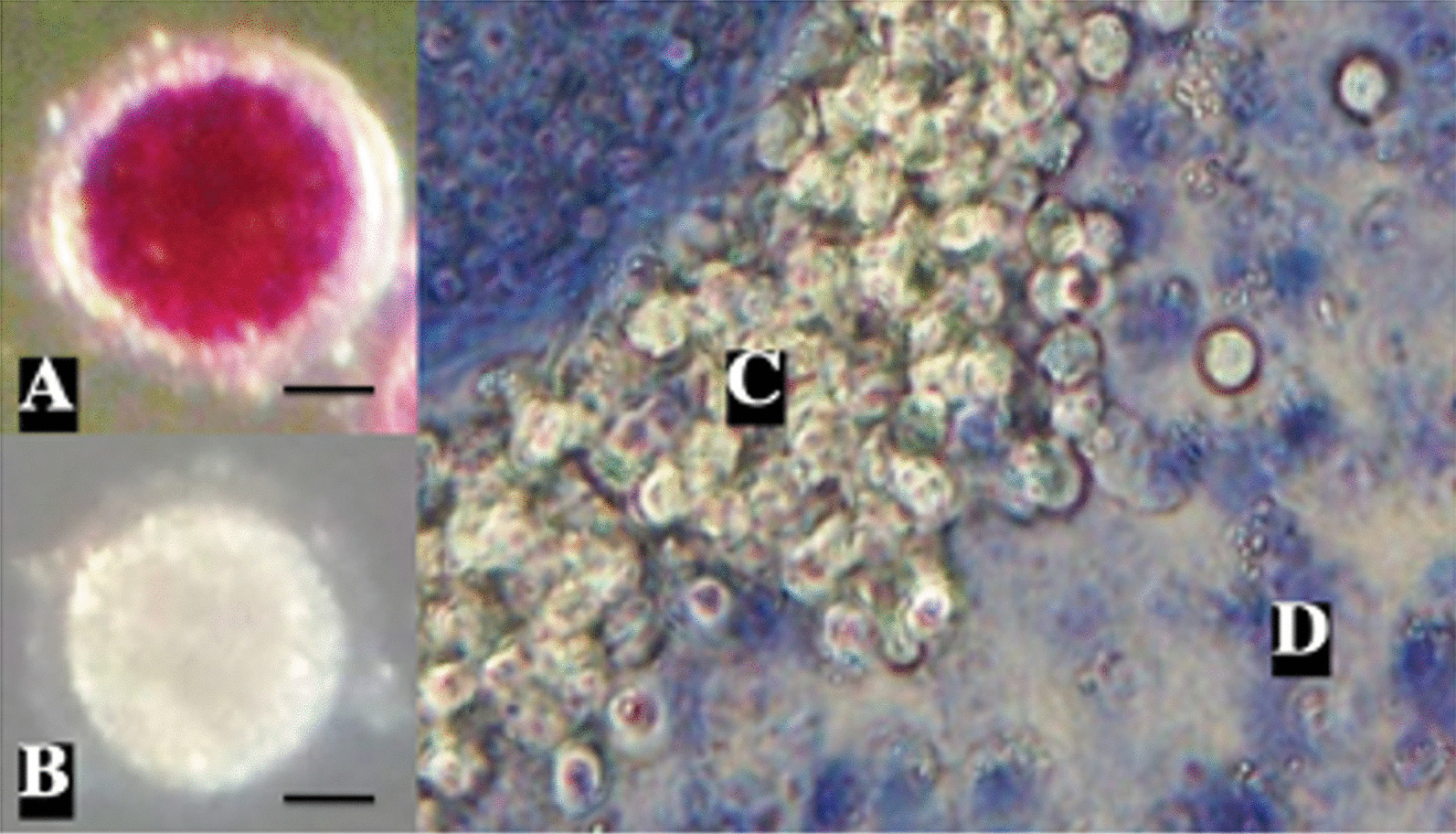
Viability evaluation in oocytes and cumulus cells. Representative images from oocytes (**a**, **b**) and cumulus cells (**c**, **d**) for viability evaluation in different groups at 40×. Oocyte (n = 304) and cumulus cells (n = 400) viability was evaluated after 44 h of in vitro maturation. For oocytes and cumulus cells, different staining agents were used: methyl-thiazolyl-tetrazolium (MTT) and trypan blue, respectively. **a** Stained purple oocyte: alive; **b** unstained oocyte: dead; **c** unstained cumulus cells: alive; **d** stained cumulus cells: dead. Scale bar: 30 μm. n = number of evaluated cells

After treatments, COCs were denuded mechanically to remove the CCs and evaluate both cell types separately. Oocytes were transferred to a 100 μL drop of 0.5 mg/mL methyl-thiazolyl-tetrazolium (MTT) diluted in modified Tris-buffered medium for viability evaluation. After 90 min, oocytes were observed under a light microscope (Zeiss Axiostar). Oocytes with purple coloration were considered as viable (Fig. 1a) and colorless ones as non-viable (Fig. 1b). For CCs viability, another agent was used. 10 μL of maturation medium with the CCs were transferred to a 10 μL drop of trypan blue. This 20 μL drop was settled in a Neubauer chamber for observation under a light microscope. Colorless cells were considered as viable and those with blue coloration as non-viable (Fig. 1c, d).

### Cryoprotectants exposure

After in vitro maturation (IVM), groups of eight to ten COCs were exposed to the highest CPAs concentration solution (10 μL) containing TCM-199 with Earle´s Salts without HEPES, 16% DMSO, 16% EG and 0.4 M sucrose at 38.5 °C for 1 min (Toxicity group). Immediately, COCs were recovered and washed three times in TL-HEPES-PVA medium. Finally, the comet assay was performed only in CCs. The CPAs concentration, exposure time and temperature were selected to make them comparable to values commonly used for oocyte vitrification protocols [9].

### Vitrification and warming

After IVM, COCs were exposed to the first vitrification solution in a four-well dish (500 μL) containing TCM-199, 7.5% DMSO and 7.5% EG for 3 min, and for 1 min in a second vitrification solution (10 μL) containing TCM-199, 16% DMSO, 16% EG and 0.4 M sucrose at 38.5 °C solution temperature. Groups of eight to ten COCs were loaded into the Cryolock, then immediately plunged horizontally into liquid nitrogen − 196 °C and stored for 30 min [9]. For warming, the Cryolock was submerged vertically in a four-well dish containing 800 μL of TCM-199 at 38.5 °C solution temperature supplemented with 0.13 M sucrose for 5 min. COCs were washed three times in phosphate buffer solution (PBS) and denuded mechanically to obtain only the CCs in order to perform the comet assay.

### DNA damage in cumulus cells by the comet assay

The DNA damage generated by CPAs exposure and vitrification in CCs was evaluated by the alkaline comet assay following the protocol by Einaudi et al. [36]. Results were expressed as Comet Tail Length (CTL) and Olive Tail Moment (OTM), then analyzed with the ChromaGen program (ODP, México). Low melting point (0.5%) and normal melting point agarose (0.1%) were prepared in PBS magnesium salt-free. Frosted slides were covered with normal melting point agarose until solidification at room temperature for at least 24 h. CCs were dissolved in low melting point agarose and added to a slide previously treated with normal melting point agarose in darkness for 10 min until solidification. Another layer of low melting point agarose was added, and immediately covered by a coverslip until solidification. Slides were immersed in a lysis solution containing 2.5 M NaCl, 100 mM Na_2_EDTA, 10 mM Tris–HCl (pH 10), 1% Triton X-100 and 10% DMSO at 4 °C for 24 h; then placed in horizontal electrophoresis and equilibrated in the buffer solution for 15 min; afterward, electrophoresis was performed at 25 V, 300 mA for 15 min. After electrophoresis, slides were placed in a neutralization solution 0.4 M Tris–HCl (pH 7.5) for 10 min. Then submerged in 70% ethanol for 5 min and, finally, dried at room temperature, for approximately 3 h. To assess DNA damage*,* slides were stained with 25 mL of ethidium bromide for 10 min [37] and analyzed using an epifluorescence microscope (Zeiss Axiostar) with the red filter (band-pass filter, 515–560 nm; long-pass filter, 590 nm), observing comets at 400×. Comet pictures were analyzed with the ChromaGen program, considering the CTL in micrometers. The CTL refers to the extent of the DNA damage. The percentage of DNA integrity refers to less DNA damage. Approximately < 15 μm of CTL is related to undamaged DNA, 15–30 μM medium damaged, and > 30 μm strong damaged. The OTM = (% tail DNA x tail length)/100.

### In vitro fertilization and embryo development

To determine the importance of the CCs during IVF and ED after warming, oocytes were fertilized in the absence (− CCs) or presence (+ CCs) of the CCs. IVF and ED were carried out following the protocol described by Casillas et al. [9]. Briefly, in vitro matured oocytes were washed twice in 500μL of TCM-199 medium and later in 500 μL of modified Tris-buffered medium (mTBM). Groups of 30 oocytes from all groups were placed into a four-well dish with 50 μL drops of mTBM covered with mineral oil and incubated for 45 min. The semen sample was obtained from one Landrace boar using the gloved hand method at a commercial insemination center, diluted in Duragen (Magapor, México) 1:2 (v:v), then transported to the laboratory at 16 °C within 2 h after collection. Sperm motility was evaluated; only semen samples with > 80% motile spermatozoa were used. Evaluation of sperm viability was performed by observation of the sample under an optical microscope. For IVF, 5 mL of the semen sample were diluted with 5 mL of Dulbecco’s phosphate buffered saline (DPBS; In Vitro, S.A., México) medium supplemented with 0.1% BSA fraction V, 75 μg/mL potassium penicillin G and 50 μg/mL streptomycin sulfate. The suspension was centrifuged (61×g for 5 min). The pellet was discarded and 5 mL of the supernatant were diluted 1:1 (v:v) with DPBS and centrifuged (1900×g for 5 min). The supernatant was discarded, and the pellet was diluted with 10 mL of DPBS and centrifuged twice under the same conditions. Later, the pellet was diluted in 100 μL of mTBM to obtain the final sperm concentration (5 X 10^5^ spermatozoa/mL). After dilution, 50 μL of the suspension were added to the medium containing oocytes, and gametes were co-incubated in mTBM for 6 h. After co-incubation, 30 putative zygotes were transferred to four-well dishes containing 500 μL drops of North Carolina State University medium (NCSU-23). ED was evaluated under an inverted microscope at 48 h (2 days post-IVF) and 168 h (7 days post-IVF). To evaluate IVF, oocytes were stained with 10 μg/mL bisbenzimide (Hoechst 33342) diluted in PBS for 40 min. The oocytes were fixed with 2% glutaraldehyde and mounted in a PBS-glycerol solution (1:9). Putative zygotes were analyzed under an epifluorescence microscope (Zeiss Axiostar) at 400 X magnification. Fertilization was assessed 16 h after IVF by visualizing pronucleus (PN) formation by the Hoechst staining method. The embryo cleavage (number of zygotes cleaved per total cultivated) and blastocyst rates (number of blastocysts per total cultivated) were determined at 48 h (2 days post-IVF) and 168 h (7 days post-IVF), respectively, by morphological evaluation under an inverted microscope (Olympus-Optical).

### Statistical analysis

Seven replicates were performed for all experiments. The data obtained from oocyte and CCs viability, DNA damage in CCs, and oocyte fertilization, cleavage, and blastocyst rates were treated as non-parametric and then analyzed by one-way analyses of variance (ANOVA) followed by a post-hoc multiple comparison Duncan test with a confidence level of *P* < 0.05 using the NCSS^11^ program. Data are presented as Mean ± SD.

## Results

In the present study, all oocytes were matured in vitro. Results indicate that the percentage of control oocytes that reached the MII stage was 73 ± 8.47% (Table 1).

**Table 1:** In vitro maturation of porcine oocytes

Maturation (mean ± SD)			
Control	GV	MI	MII
	44/369 (12 ± 1.51)	57/369 (15 ± 8.81)	268/369 (73 ± 8.47)

Porcine oocytes were matured in vitro for 44 h and Hoechst stain was performed to evaluate oocyte maturation stages in control (n = 369 evaluated oocytes). Oocytes in GV and MI, were considered immature and oocytes in MII as matured. Data are presented as mean ± standard deviation (SD)

GV = germinal vesicle; MI = metaphase I; MII = metaphase II

### Oocyte and cumulus cells viability after CPAs exposure and vitrification

**Fig. 2:**
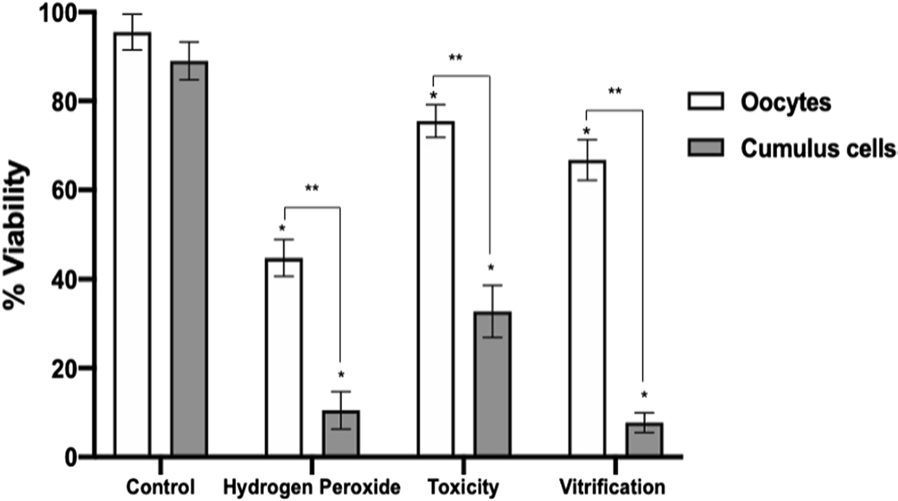
Percentage of oocyte and cumulus cells viability. Hydrogen peroxide (COCs exposed to 2.2% H_2_O_2_), Toxicity (16% EG + DMSO), and Vitrification (EG + DMSO + Vitrification). Cumulus cells (CCs) were removed from oocytes (n = 304) for evaluation. Control (n = 242 evaluated CCs), H_2_O_2_ (n = 263 evaluated CCs), Toxicity (n = 204 evaluated CCs), Vitrification (n = 219 evaluated CCs). In all groups, decreased viability was observed in both cell types compared to control. Compared to oocytes, CCs viability decreased significantly in all groups. Data are presented as mean ± standard deviation (SD). Significant differences were considered when *P* < 0.0001. *Indicates significant difference versus control. **Indicates significant difference between oocytes and cumulus cells. H_2_O_2_ = hydrogen peroxide; EG = ethylene glycol; DMSO = dimethylsulphoxide. n = number of evaluated cells

Viability was evaluated in oocytes and CCs separately after IVM by staining in all groups (Figs. 1, 2). COCs treated with H_2_O_2_ were used as a positive control. Results demonstrate that viability after CPAs exposure Toxicity group (75.5 ± 3.69%, oocytes; 32.7 ± 5.85%, CCs) and vitrification (66.7 ± 4.57%, oocytes; 7.7 ± 2.21%, CCs) was significantly lower (*P < 0.001) in both cell types compared to control (95.5 ± 4.04%, oocytes; 89 ± 4.24%, CCs) (Fig. 2). CCs viability was significantly reduced (*P < 0.001) after vitrification compared to control (7.7 ± 2.21% vs. 89 ± 4.24%, respectively). Compared to oocytes, CCs viability decreased significantly in all treatment groups (**P < 0.0001).

### Cumulus cells DNA damage after CPAs exposure and vitrification

**Fig. 3:**
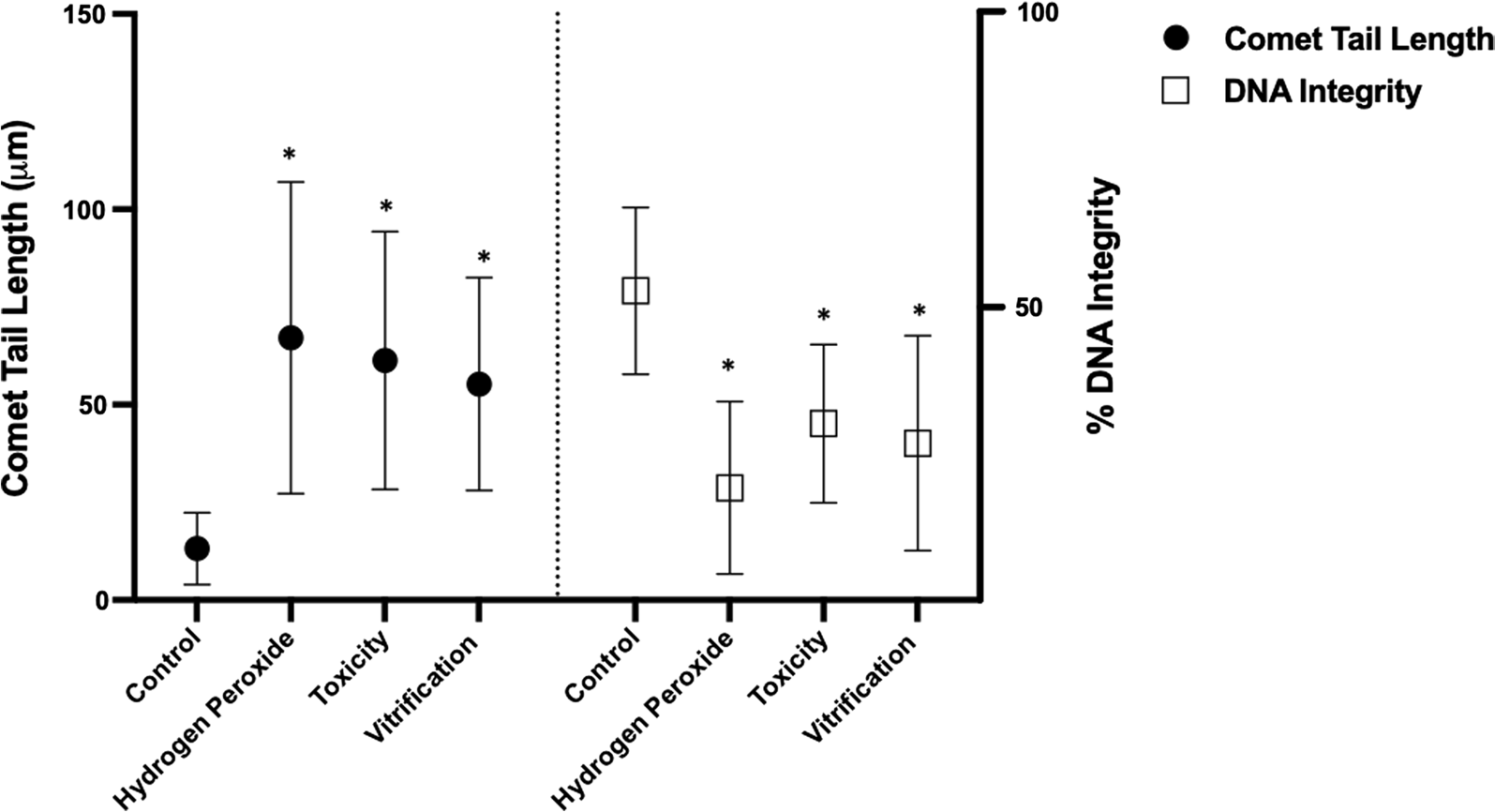
Cumulus cells genotoxicity assessment by the comet assay expressed by the Comet Tail Length (CTL) and DNA Integrity. Hydrogen peroxide (COCs exposed to 2.2% H_2_O_2_), Toxicity (16% EG + DMSO), and Vitrification (EG + DMSO + Vitrification). Cumulus cells (CCs) were removed from oocytes for evaluation. Control (n = 242 evaluated CCs), H_2_O_2_ (n = 263 evaluated CCs), Toxicity (n = 204 evaluated CCs), Vitrification (n = 219 evaluated CCs). The CTL refers to the extent of DNA damage, and DNA integrity to the percentage of DNA in the comet’s head (no DNA damage). Approximately < 15 μm of CTL is related to normal or undamaged DNA, and damaged > 30 μm. Higher CTL and lower DNA integrity was obtained in all groups compared to control. Data are presented as mean ± standard deviation (SD). Significant differences were considered when *P* < 0.05. *Indicates significant difference versus control. CTL = comet tail length; H_2_O_2_ = hydrogen peroxide; EG = ethylene glycol; DMSO = dimethylsulphoxide

In the comet assay, fragmented DNA shows the characteristic appearance of a comet tail, while undamaged DNA appears as an intact head. Results demonstrate that higher (*P < 0.05) CTL was obtained in all groups (67.1 ± 39.88 μm, H_2_O_2_; 61.2 ± 32.98 μm, Toxicity; 55.2 ± 27.21 μm, Vitrification) compared to control (13.1 ± 9.22 μm) (Fig. 3; filled circle). Also, results indicate that the percentage of DNA integrity (less DNA damage) was significantly reduced (*P < 0.05) in all groups (28.7 ± 22.09%, H_2_O_2_; 45.1 ± 20.29%, Toxicity; 40.1 ± 27.52%, Vitrification) compared to control (79.1 ± 21.33%) (Fig. 3; empty square).

**Fig. 4:**
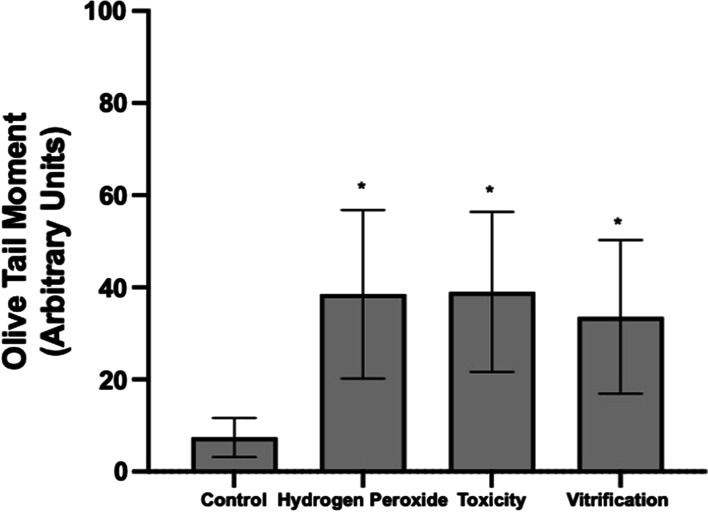
Cumulus cells genotoxicity assessment by the comet assay expressed by the Olive Tail Moment (OTM). Hydrogen peroxide (COCs exposed to 2.2% H_2_O_2_), Toxicity (16% EG + DMSO), and Vitrification (EG + DMSO + Vitrification). Cumulus cells (CCs) were removed from oocytes for evaluation. Control (n = 242 evaluated CCs), H_2_O_2_ (n = 263 evaluated CCs), Toxicity (n = 204 evaluated CCs), Vitrification (n = 219 evaluated CCs). The OTM represents the product of the percentage of total DNA in the tail and the distance between the centers of the head and tail regions. High OTM value indicates DNA damage. Higher OTM values were obtained in all groups compared to control. Data are presented as arbitrary units. Significant differences were considered when *P* < 0.05. *Indicates significant difference versus control. OTM = olive tail moment; H_2_O_2_ = hydrogen peroxide; EG = ethylene glycol; DMSO = dimethylsulphoxide

**Fig. 5:**
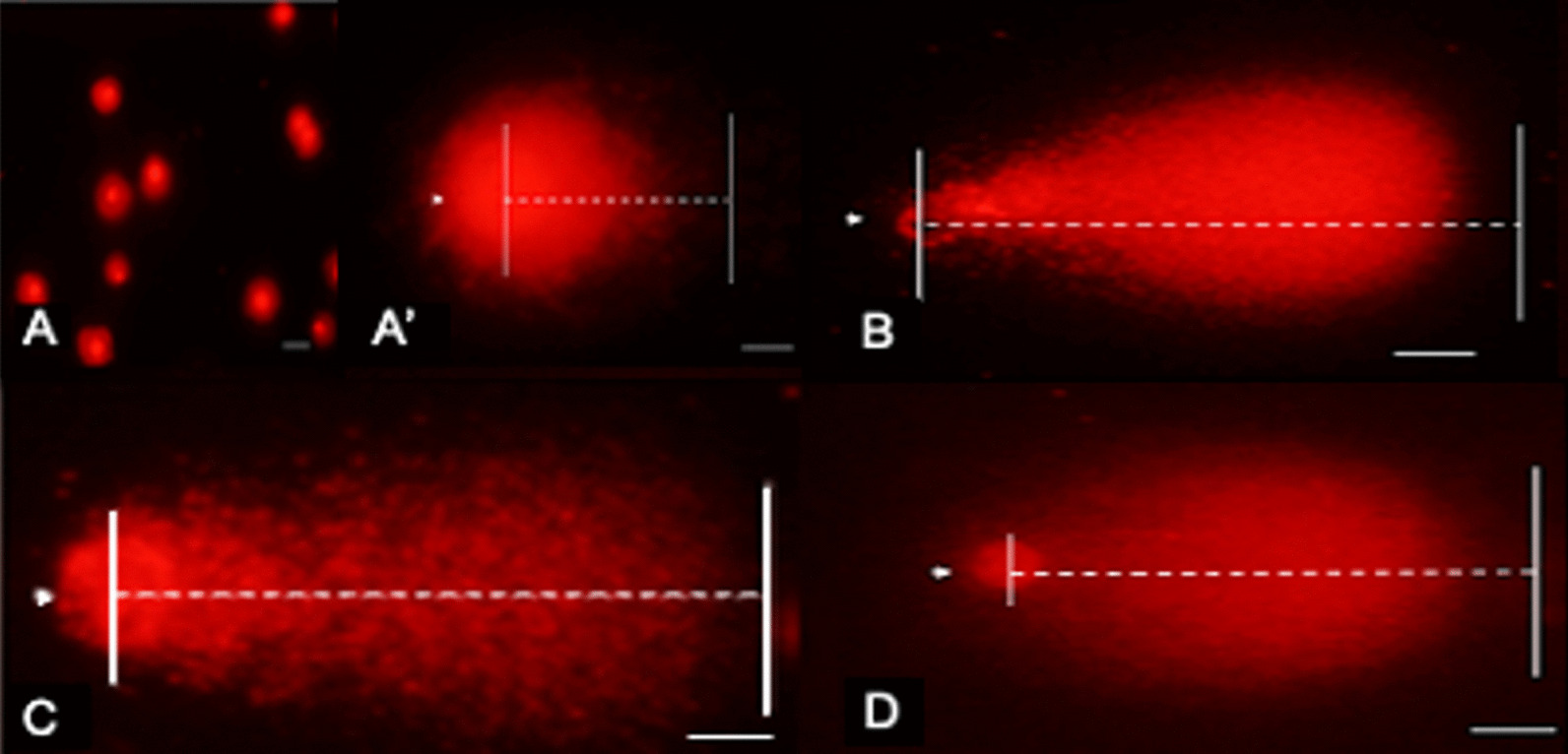
Cumulus cells comet assay evaluation. Representative images of comet assay evaluation. The direction of electrophoresis was left to right, and DNA fragments are observed as a comet tail. **a** Cumulus cells control magnification at 200×; no DNA migration. Scale bar: 15 μm. **a**’ Cumulus cells control: one cell magnification at 400×; no DNA migration. Scale bar: 15 μm. **b** Cumulus cells exposed to H_2_O_2_ magnification at 400×; DNA migration. Scale bar: 15 μm. **c** Cumulus cells exposed to EG + DMSO magnification at 400×; DNA migration. Scale bar: 15 μm. **d** Cumulus cells EG + DMSO + Vitrification group magnification at 400×; DNA migration. Dotted line indicates the CTL and the arrowhead the nucleoid of the cumulus cell. The percentage of DNA integrity is presented as less DNA damage, shown in **a** and **a**’ pictures

The DNA damage in CCs was also measured using the alkaline comet assay and expressed as OTM. The OTM = (% tail DNA x tail length)/100. In terms of the OTM, results indicate that H_2_O_2_, Toxicity, and Vitrification groups (38.5 ± 18.30, 39 ± 17.41, 33.6 ± 16.69, respectively) were significantly higher (*P < 0.05) than control (7.4 ± 4.22), demonstrating that higher DNA damage is produced after CPAs exposure and vitrification (Figs. 4, 5).

### Oocyte in vitro fertilization and embryo development after CPAs exposure and vitrification in the absence or presence of cumulus cells

**Fig. 6:**
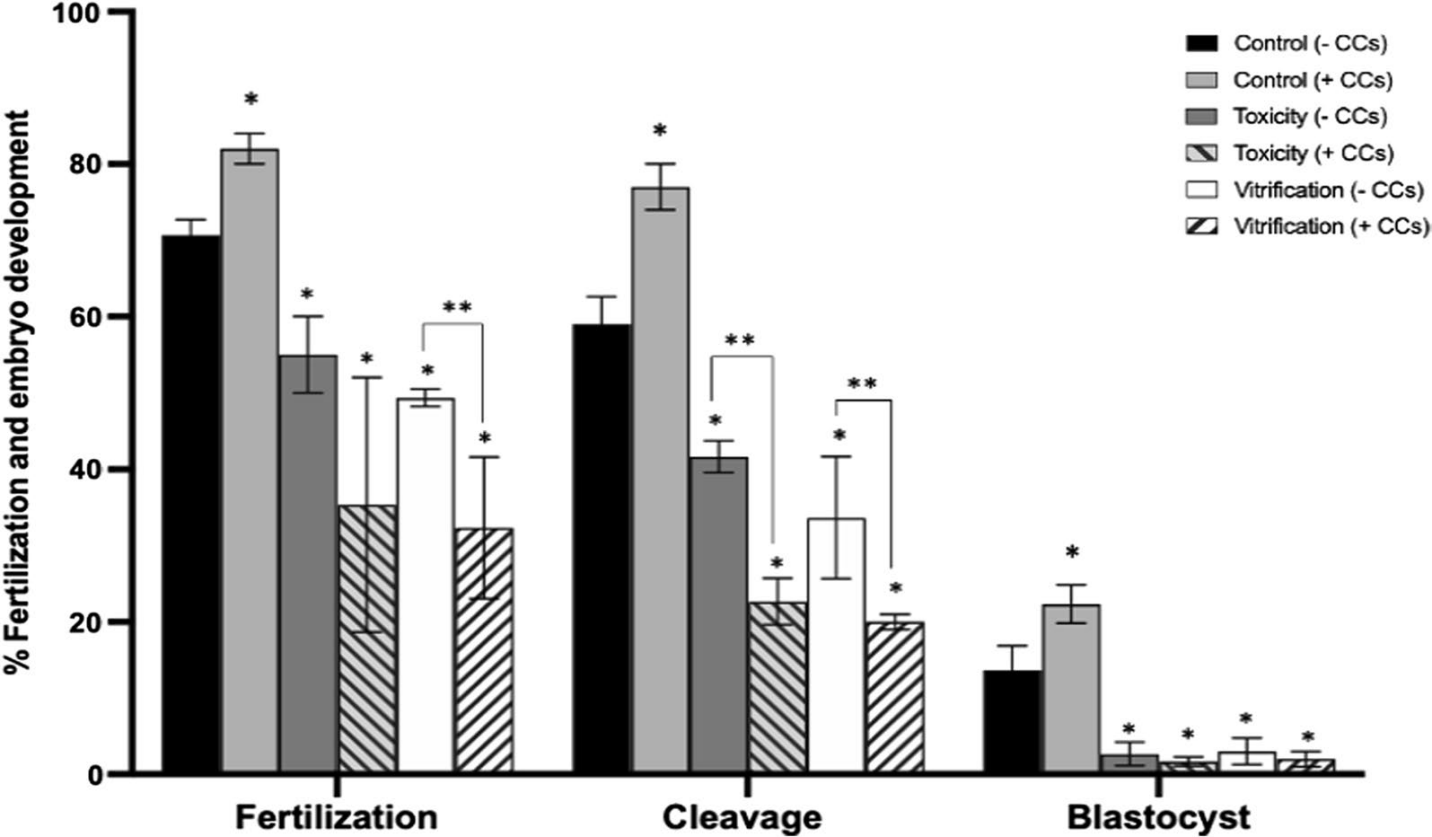
In vitro fertilization, cleavage, and blastocyst rates evaluation. Toxicity (16% EG + DMSO), and Vitrification (EG + DMSO + Vitrification). After treatments, to determine the importance of the cumulus cells during fertilization and embryo development, oocytes were fertilized in the absence (− CCs) or presence (+ CCs) of the CCs. The number of evaluated oocytes/treatment was n = 214. Oocyte fertilization, cleavage, and blastocyst rates increased significantly with the presence (+ CCs) of CCs compared to denuded oocytes in control groups. However, fertilization, cleavage and blastocyst rates significantly decreased in the Toxicity and Vitrification groups. Also, the presence of CCs in the toxicity and vitrification groups did not increase fertilization, cleavage, and blastocyst rates compared to control. Data are presented as mean ± standard deviation (SD). Significant differences were considered when *P* < 0.0001. *Indicates significant difference versus the respective control. **Indicates significant difference between treatments. EG = ethylene glycol; DMSO = dimethylsulphoxide. n = number of evaluated cells

Results demonstrate that oocyte fertilization (70.6 ± 2.08% vs. 82 ± 2%), cleavage (59 ± 3.60% vs. 77 ± 3%), and blastocyst rates (13.6 ± 3.21% vs. 22.33 ± 2.51%) was significantly higher in granulose-intact oocytes (+ CCs) compared to denuded oocytes (− CCs) in control groups (*P < 0.0001). Additionally, fertilization, cleavage and blastocyst rates significantly decreased in granulose-intact oocytes in the Toxicity (35.3 ± 16.65%, 22.6 ± 3.05%, 1.6 ± 0.57%, respectively) and Vitrification (32.3 ± 9.29%, 20 ± 1%, 2 ± 1%, respectively) groups compared to control (82 ± 2%, 77 ± 3%, 22.3 ± 2.57%, respectively) (*P < 0.0001) (Fig. 6).

## Discussion

In different mammalian species including humans and swine, the preservation of intact CCs after COCs vitrification is of great importance because these cells play important roles in the maturation and fertilization processes [38, 39]. However, most studies have been carried out to evaluate the effects caused by vitrification on the oocytes leaving aside the importance of the CCs [28–30].

### Effect of vitrification on cumulus cells and oocyte viability

Our results demonstrate that CPAs exposure (toxicity group) and vitrification decreased CCs and oocyte viability. Moreover, the decrease in viability after vitrification was greater in CCs compared to oocytes (7.7 ± 2.21% vs. 66.7 ± 4.57%, respectively). In the present study, the decrease in viability in both cell types may be mainly due to the toxicity of CPAs, as these substances are used at high concentrations in vitrification protocols. Gurtovenko and Anwar [40] reported the possible mechanism of the interaction of several of the most used CPAs with the lipid bilayer. DMSO has a greater ability to diffuse across the phospholipid bilayer than EG. DMSO at high concentrations (40%) can destroy cell membranes completely [40]. The sulfinyl oxygen binds to water strongly and DMSO can surround polar head groups of cell membranes, which may help explain the compound toxicity [41]. In agreement with the results obtained in the present study, it was previously reported that the use of EG affects CCs survival after freezing. Surprisingly, even though EG is widely used for embryo cryopreservation, low survival rates in CCs are reported [32]. According to the literature, toxicity is reduced by combining CPAs [41, 42]. Therefore, in the present study, we used EG + DMSO for vitrification since it has been proven that this mixture allows high survival rates in ewe oocytes [42] and porcine embryos [43]. Another study with porcine oocytes reported that EG + DMSO and EG + PROH resulted in similar viability and IVM rates after vitrification [44]. Somfai et al. [2] reported that the mixture of EG + DMSO allows the production of viable blastocysts after immature oocyte vitrification. Also, we previously reported that EG + DMSO resulted in a 30% blastocyst formation [9]. However, high concentrations of CPAs are still used (16–50%), promoting detrimental effects in cells, especially DNA damage either in the CCs or the oocytes [20, 28, 31, 32, 37, 45–47]. For example, it was reported that DMSO inhibits CCs expansion in a concentration-dependent manner, resulting in cell death by apoptosis [48]. EG has been used as an effective cryoprotectant for bovine oocytes [49] and embryos [50]. El-Shanat et al. [49] reported that immature buffalo oocytes vitrified with EG or DMSO resulted in 85 and 83% of morphological normal oocyte-CCs, respectively. However, maturation rates were low (47% when vitrified with EG and 46% when vitrified with DMSO). One study evaluated the viability of ewe oocytes after vitrification with different cryoprotectants. They reported that oocyte viability was higher (88.16%) when using (17% EG + 17% DMSO mixture) compared to (70.95%) with (34% EG) or (68.76%) with (17% EG + 17% PROH mixture) [42]. However, most studies evaluate the viability of the oocytes after vitrification but not that of the CCs. In this regard, compared to oocytes, the CCs are smaller in size, and are the first in contact with the CPAs protecting the oocytes during vitrification, which implies that high CPAs concentrations are initially received by these cells, producing greater cytotoxic damage. Accordingly, the results obtained in the present study indicate that CCs do protect oocytes after vitrification; however, most of them lose their viability. In agreement, other study reported that the CCs protect and promote cumulus enclosed MII oocyte survival after vitrification in equine oocytes [20]. Tharasanit et al. [20] reported that the proportion of dead CCs after the vitrification of GV oocytes with 10% EG + 10% DMSO equilibration solution, and 20% EG + 20% DMSO and 0.5 M sucrose vitrification solution was 13.7% and CPAs exposure without vitrification was 2.7%. When evaluating the viability of the CCs in oocytes that reached maturation (MII stage) an even greater decrease in viability was observed (36%). It was also reported that CCs are more affected than oocytes after vitrification since membrane damage is produced in mouse GV oocytes [51].

In the present study, MII oocyte viability diminished up to 66.7 ± 4.57% after vitrification. In this regard, the nuclear cell stage before vitrification is another key factor to be considered. GV [52] or MII oocytes have fewer CPAs and water permeability than zygotes and later-stage embryos [53]. The vitrification of denuded MII oocytes could generate alterations in the plasma membrane, mitochondrial distribution, meiotic spindle, and chromosomes [54]. Rojas et al. [28] reported that vitrified MII oocytes show spindle abnormalities because chromosomes are exposed directly to CPAs. When oocytes are vitrified with the CCs, these cells can prevent oocyte cryodamage [21]. In the present study, the reduction of oocyte viability up to 75.5 ± 3.69% after CPAs exposure and 66.7 ± 4.57% after vitrification could be due to the possible oocyte injuries caused by the CPAs as already mentioned above.

### Effect of vitrification on cumulus cells DNA integrity

Results demonstrate that CPAs exposure and vitrification generated DNA damage in CCs. According to the literature, little is known about the DNA damage generated after vitrification in porcine CCs and most studies only evaluate the cryoinjuries produced in oocytes [55]. In this regard, it was reported that the use of 20% of EG + 20% DMSO produced DNA damage in porcine vitrified GV oocytes, where 54.8% of oocytes resulted in DNA damage compared to 5.6% in the control group [55]. The DNA damage in CCs may be generated since the concentrations of CPAs used during vitrification are very high for this cell type. Generally, in most vitrification protocols, these concentrations are calculated considering the characteristics of the oocytes but not those of the CCs. Therefore, this may cause the CCs to suffer more damage by vitrification than the oocytes. In agreement, Taghizabet et al. [21], reported that CCs create a natural protective shield around the oocyte against physico-chemical insults due to vitrification. In addition, the DNA damage generated in CCs after vitrification could also be due to the production of reactive oxygen species (ROS) [56, 57]. For example, H_2_O_2_ is believed to cause DNA strand breaks after conversion to the hydroxyl radical [37]. Accordingly, H_2_O_2_ was used in the present study as DNA damage-inducer (positive control).

For the evaluation of cell genotoxicity caused by CPAs exposure and vitrification, the comet assay has generally been used as an evaluation method. Most studies consider the CTL as an indicator of the damage extent [58] and the percentage of DNA as fragmentation; however, the OTM is considered the most reliable value [59]. The CTL is related to the percentage of DNA integrity as high CTL values indicate less DNA integrity. In the present study, an alkaline comet assay was performed to detect different types of DNA lesions including single (SSBs) and double-strand breaks (DSBs). The SSBs represent the most common type of DNA damage and unrepaired SSBs can alter DNA replication and transcription, resulting in diseases [60]. In contrast, DSBs are one of the most severe forms of DNA damage, and can cause cell death, chromosome aberrations or loss of genetic material. However, one of the limitations of this study is that the alkaline version does not allow simultaneous discrimination between SSBs and DSBs.

### Effect of vitrification on fertilization and embryo development in the presence or absence of cumulus cells

In porcine oocytes, more studies are needed since ED rates after vitrification are still reported to be low [9, 61, 62]. In the present study, the CCs were not removed from vitrified oocytes to evaluate their importance during fertilization, cleavage and ED. Results demonstrate that oocyte fertilization, cleavage, and blastocyst rates increased with the presence of the CCs compared to denuded oocytes in control groups. In vitro studies reported that CCs removal decreases fertilization rates in humans [63], and pigs [64]. Also, another study in porcine oocytes reported that the presence of CCs during IVF has a positive influence on ED [65]. However, in CPAs exposed and vitrified oocytes, fertilization, cleavage, and ED rates significantly decreased compared to control. This fact could be explained by the results obtained in the present study, in which the decreased CCs viability and the generation of DNA damage after vitrification, could affect CCs-sperm recognition prior to fertilization. In this regard, Dos Santos-Neto et al. [66] suggested to avoid CCs removal before IVF in sheep MII oocytes and the addition of a fresh CCs co-culture system for improving blastocyst production. This suggests that during vitrification the CCs protect the oocyte but for the subsequent processes it is necessary to replace damage cells with intact ones to ensure better ED rates. They reported that vitrification of MII oocytes, fertilized with CCs resulted in 22% cleavage rate, and 9.2% blastocyst rate. In matured oocytes without CCs, cleavage resulted in 15.1% and blastocyst rate in 4.6%. In the present study with porcine oocytes, we obtained 33.6 ± 8.02% cleavage (− CCs) and 3 ± 1.73% blastocyst rate (− CCs) compared to 20 ± 1% cleavage (+ CCs) and 2 ± 1% blastocyst (+ CCs) [66]. Results obtained in the present study were similar to those reported in sheep oocytes regarding cleavage and blastocyst rates when fertilized with or without CCs [66]; however, differences between species should be considered. Therefore, we suggest that the vitrification of porcine mature oocytes should be carried out without removing the CCs since a higher oocyte viability is obtained. However, since viability in the CCs is significantly reduced, the use of a co-culture system with fresh intact CCs after vitrification could increase IVF and ED rates. In agreement, Dos Santos-Neto et al. [66] reported that the addition of a co-culture system with CCs increases blastocyst rates up to 10.7% in sheep. Also, it was previously reported that, in the case of vitrified porcine immature oocytes, these cells can be used in co-culture systems improving IVM [23], cleavage and blastocyst rates [22, 62].

CCs are important in all processes of oocyte development from maturation [39] to embryo development. The CCs can prevent premature exocytosis of cortical granules as well as the hardening of the zona pellucida to avoid failure of sperm-oocyte recognition, allowing fertilization [15]. It has also been reported that *HAS2*, *VCAN* and progesterone receptor mRNA expression is increased in CCs associated with oocytes that have reached the blastocyst stage [18]. Therefore, the results obtained in the present study strongly suggest that CCs integrity after CPAs exposure and vitrification is an important factor to be considered for further oocyte developmental competence.

## Conclusions

This study demonstrates that oocyte exposure to CPAs or vitrification reduced viability in oocytes and CCs, and generated DNA damage in the CCs, affecting fertilization and ED rates. The decline in oocyte fertilization, cleavage, and blastocyst rates after CPAs exposure or vitrification can be attributed to the reduction in both cell types viability, and the generation of DNA damage in the CCs. These findings will allow to understand some of the mechanisms of oocyte damage after vitrification that compromise their developmental capacity, as well as the search for new vitrification strategies to increase fertilization and ED rates by preserving the integrity of the CCs.

## Data Availability

Not applicable.

## Abbreviations

CC: Cumulus cells
COCs: Cumulus-oocyte complexes
CPAs: Cryoprotectant agents
CTL: Comet tail length
DMSO: Dimethylsulfoxide
ED: Embryo development
EG: Ethylene glycol
EGF: Epidermal growth factor
GV: Germinal vesicle
ICSI: Intracytoplasmic sperm injection
IVF: In vitro fertilization
IVM: In vitro maturation
MI: Metaphase I
MII: Metaphase II
MTT: Methyl-thiazolyl-tetrazolium
OTM: Olive tail moment
PG: Propylene glycol
PROH: 1,2-Propanediol
PVA: Polyvinyl alcohol
ROS: Reactive oxygen species
